# Demethylation drug 5-Aza-2’-deoxycytidine-induced upregulation of miR-200c inhibits the migration, invasion and epithelial-mesenchymal transition of clear cell renal cell carcinoma *in vitro*

**DOI:** 10.3892/ol.2021.12923

**Published:** 2021-07-14

**Authors:** Juan Jiang, Bo Yi, Siqing Ding, Jian Sun, Wei Cao, Mengzi Liu

Oncol Lett 11: 3167-3172, 2016; DOI: 10.3892/ol.2016.4364

Following the publication of this paper, it was drawn to the Editors’ attention by a concerned reader that certain of the scratch-wound assay data shown in Fig. 3A, and the Transwell cell migration assay data in Fig. 4, were strikingly similar to data appearing in different form in other articles by different authors. Owing to the fact that the contentious data in the above article had already been published elsewhere, or were already under consideration for publication, prior to its submission to *Oncology Letters*, the Editor has decided that this paper should be retracted from the Journal. The authors were asked for an explanation to account for these concerns, but the Editorial Office did not receive any reply. The Editor apologizes to the readership for any inconvenience caused.

